# Factors Influencing University Students’ Persistence and Satisfaction Towards Self-Directed Language Learning Using Mobile Technology

**DOI:** 10.3390/bs16040519

**Published:** 2026-03-30

**Authors:** Yuzhi Lai, Nadira Saab, Wilfried Admiraal

**Affiliations:** 1 School of Foreign Studies, Hefei University of Technology, Hefei 230601, China; 2 ICLON, Leiden University Graduate School of Teaching, Leiden University, Kolffpad 1, 2333 BN Leiden, The Netherlands; n.saab@iclon.leidenuniv.nl; 3 Centre for the Study of Professions, Oslo Metropolitan University, St. Olavs Plass, P.O. Box 4, N-0130 Oslo, Norway; wilfried@oslomet.no

**Keywords:** self-directed learning, mobile technology, language learning, structural equation modeling, persistence, higher education

## Abstract

Research on mobile-assisted language learning has mainly focused on teacher-initiated learning, instead of student-initiated learning outside of class. In self-directed language learning with mobile technology, students’ satisfaction with and persistence in learning are conditionafor making self-directed learning effective. This study examined how university learners’ persistence and satisfaction towards self-directed language learning using mobile technology are predicted by mobile readiness, teacher support, and engagement. Survey data from 446 language learners in different disciplines attending Chinese universities were analyzed using structural equation modeling. Learners’ satisfaction was found to be significantly and positively related to their mobile readiness and persistence to both mobile readiness and engagement. Additionally, learners’ mobile readiness was found to make a strongly significant contribution to engagement in self-directed learning using mobile technology. And teacher support was significantly and positively linked to learners’ mobile readiness yet negatively to learners’ engagement. However, the findings showed an indirect and positive impact on learners’ engagement with a mediating role for mobile readiness. Considering the importance of learners’ mobile readiness and the critical impact of teacher support in our context, further research should explore learners’ characteristics and teacher support in mobile self-directed learning settings.

## 1. Introduction

The proliferation of mobile technologies has generated fresh prospects for language learning, ensuring its widespread availability, ease of access, and adaptability (Hafour, 2022; Hsu & Lin, 2022). Over the past few years, research on mobile-assisted language learning has been on the rise. For example, Loewen et al. (2019) investigated university students’ language learning experiences and results of learning Turkish on Duolingo in the United States. Y. Wang et al. (2021), in Australia, probed students’ perceptions about Chinese Island (CI), an immersive 3D virtual environment, to engage Chinese language learning students, facilitate their authentic language use, and enhance their learning experience. van Lieshout and Cardoso (2022) examined the potential of Google Translate as a pedagogical tool to learn Dutch language phrases and associated pronunciation. To date, nevertheless, the majority of mobile-assisted language learning research has focused on teacher-initiated learning (e.g., C. Gao & Shen, 2021; Ghorbani & Golparvar, 2020; Lee et al., 2017; Tai, 2022), instead of student-initiated learning outside of class using mobile technology (An et al., 2020). In some nations, university students are not given enough time within the language curriculum to practice and acquire foreign languages in class (Liu, 2020; Trinder, 2017). To tackle this issue, Y. Lai et al. (2022b) and Pramesti (2020) suggested that students conduct self-directed and out-of-class language learning assisted by mobile technology to frequently expose themselves to authentic language environments, thereby further maximizing their language abilities. According to Loyens et al. (2008), self-directed learning is different from self-regulated learning, although some researchers use both terms interchangeably. Both terms involve the active engagement and goal-directed behavior of students but differ in the degree of control the learners have, particularly at the beginning of the learning process (Loyens et al., 2008): self-directed learners are the initiators of a learning task, whereas self-regulated learners work on tasks that are set by the teacher. Mobile technology in this study refers to portable electronic devices such as smartphones, tablets, and laptops, as well as the software and applications designed to be used on them. These devices allow learners to access resources on the go and communicate with others. In this self-directed language learning using mobile technology (SDLLMT) context, learners are in charge of their own language learning with the assistance of mobile technology outside the classroom, and they can determine what and how to learn (Merriam & Bierema, 2013). More explicitly, students make use of mobile applications such as Instagram, YouTube, Tandem, and Google Translate, as well as foreign language-specific apps like HelloTalk and Duolingo to set up a language learning environment.

SDLLMT is an under-researched field (An et al., 2020; Nguyen & Takashi, 2021). Yet several studies were found regarding students’ perceptions on or experiences with using mobile technology in self-directed learning (C. Lai et al., 2018; García Botero et al., 2019), their learning strategies (Y. Lai et al., 2022a), and the behavioral intention and adoption of using mobile technology in self-directed language learning (Y. Lai et al., 2022b; C. Lai, 2013; C. Lai & Zheng, 2018; Zhang & Perez-Paredes, 2021). However, research on intended outcomes, such as learners’ satisfaction and persistence, has been limited up to now. Persistence is an important outcome variable as learning success in the online environment, and in online self-directed learning, in particular, depends on learners’ perseverance of their learning activities (Joo et al., 2013). Satisfaction is an important affective outcome as well, as satisfied learners are more willing to try again and to persist in the long term (Ji et al., 2022). Despite the growing popularity of SDLLMT among students, the initial adoption of this type of learning does not ensure successfully acquiring a new language (S. Yang et al., 2019). Learners need to persevere throughout the learning process since mastering languages takes years, not a couple of days (Fryer, 2019). Researchers noted, however, that even when surrounded by teachers’ or institutional support, learners easily give up (Cheng & Lee, 2018); this might be much less the case if they rely on themselves to take complete control in self-directed learning with mobile technology. Yet a decrease in motivation or lack of useful materials could lead learners to give up this self-directed learning using mobile technology, without taking any responsibility and without consequences. For example, Luo (2019) investigated 325 Chinese university students in terms of their mobile English learning. The results showed that 51% had always used mobile technology for language learning, but most students did not use it consistently over a period, and 70% were unable to focus on language tasks using mobile devices for more than 20 min at a time. For these reasons, this study aims to investigate the factors affecting learners’ persistence and satisfaction when conducting SDLLMT. Consequently, the findings of this study might contribute to a better understanding of how to enhance learners’ outcomes in SDLLMT. This study endeavors to provide guidance for self-directed learners and teachers to encourage learners’ persistence in their SDLLMT. This study also explores the role of facilitators (teachers in this study) in self-directed learning outside class.

## 2. Literature Review

### 2.1. Learners’ Persistence and Satisfaction

Persistence, also known as continuance intention, has been regarded as a noteworthy indicator for quality evaluation in online learning (Joo et al., 2013) and an important variable for keeping students committed to the process of SDLLMT, as there is no teacher involved. Learners’ satisfaction relates to learners’ overall perceptions of their own experience when using mobile technology in their self-directed language learning (Rabin et al., 2019). It affects their motivation, which is an important psychological factor affecting student learning (Ji et al., 2022). Henderikx et al. (2017) and Reich (2014) have claimed that success in open learning contexts should be evaluated by learner-centered measures such as learner satisfaction.

Previous studies have examined factors influencing university students’ satisfaction and persistence in an online context. In mobile language learning, however, studies that investigated satisfaction and persistence are mostly related to self-regulated language learning instead of self-directed language learning (Karaoğlan Yılmaz, 2021; S. Yang et al., 2019; Huang & Yu, 2019). Only X. Wang et al. (2022) investigated student-initiated learning behavior in a mobile language context. They examined the relationship between continuance intention and perceived usefulness and the mediation effects of flow and integrative motivation. Continuance intention is represented as the repeated usage of language learning apps in the learning process. Flow refers to the optimal experience that one has while using language learning apps, which includes three dimensions: concentration, control and enjoy. Integrative motivation refers to the case of learning a language due to desire or interest to understand the target culture, and perceived usefulness indicates to what extent a user thinks that technologies can enhance performance. The results of correlation analysis indicated that the four variables were significantly and positively related to each other. Regression analysis showed that flow and integrative motivation played mediating roles in the relationship between continuance intention and perceived usefulness. Yet, this study did not address teacher support or students’ mobile readiness.

Students’ mobile readiness and teacher support are both important factors that can be related to self-directed learning outside class. Since self-directed learning using mobile technology outside class is completely initiated and controlled by the learners themselves, the learners have more autonomy. Higher learning autonomy implies that learners themselves can exert greater influence on the learning process (Kuo et al., 2021). One of the learner characteristics that affects success in mobile learning could be mobile readiness (H.-H. Lin et al., 2016). In addition, in spite of the dominant role of learners in this context, they could also seek and receive teachers’ help to support their language learning (Y. Lai et al., 2022b). C. Lai (2015) stated that teachers play a significant role in influencing students’ self-directed learning, and they can shape the quality and quantity of students’ technology use outside the classroom. In addition to teacher support and mobile readiness, learners’ engagement was included in this study. Learners’ engagement is associated with a high persistence rate and learning success (Pursel et al., 2016). Thus, it is necessary to include learners’ engagement, their mobile readiness and teacher support in this study to understand learners’ SDLLMT.

### 2.2. Learners’ Engagement

Engagement in SDLLMT refers to the ongoing time, effort, and energy that learners put into this form of independent learning to achieve their goals (Kuo et al., 2021). Student engagement has been conceptualized and operationalized as a multidimensional construct which can be broken down into behavioral, emotional, and cognitive engagement. Behavioral engagement refers to students participating in a language learning activity (Fredricks et al., 2004). Emotional engagement refers to students’ affective reactions toward their learning experience (Kuo et al., 2021). These emotional feelings include enthusiasm, enjoyment, interest, fun, boredom, and feelings of depression (Deng et al., 2020). Cognitive engagement refers to learners putting effort into understanding and mastering language knowledge and skills (Skinner et al., 2008). Ji et al. (2022) called for studies to explain how the different dimensions of language learners’ engagement are associated with satisfaction in an online language learning environment. This study was timely, therefore, as it aimed to fill this gap by investigating learner engagement as a multidimensional construct.

Researchers have reported that learner engagement influences persistence and satisfaction in online learning (J. A. Gray & DiLoreto, 2016; Jung & Lee, 2018; Shin & Sok, 2023). Shin and Sok (2023) showed a positive and significant relationship between engagement and satisfaction and perceived learning in an online second language learning environment. And Jung and Lee (2018) revealed that engagement not only had a direct effect on learning persistence but also mediated between the presence of a teacher and learning persistence in K-MOOCs. Based on these studies, we assume that learners’ engagement is linked to persistence and satisfaction and plays a mediating role in the process of their SDLLMT.

### 2.3. Mobile Readiness

Mobile learning is slightly different from online learning and e-learning, being characterized by mobility and situated learning. For this reason, H.-H. Lin et al. (2016) warned us not to adopt existing e-learning/online learning readiness scales in mobile learning studies. The present study therefore employed a mobile readiness scale, instead of the widely used online/e-learning readiness scales. Mobile readiness is defined as “an individual’s propensity to use mobile technology to execute formal and informal learning activities” (H.-H. Lin et al., 2016). More specifically, in our context, mobile readiness refers to learners’ mobile-related knowledge, attitudes, skills, and competencies in utilizing mobile technology effectively to achieve self-directed learning objectives. According to H.-H. Lin et al. (2016), mobile readiness is a three-dimensional construct, including self-directed learning, mobile learning self-efficacy, and optimism. Self-directed learning is a personality trait where learners are self-motivated and responsible for their own learning process. To distinguish it from SDLLMT, self-directed learning is renamed as SDL disposition. Learning self-efficacy defines learners’ self-perceived capability to master the functions of mobile technology and systems and to learn well using the mobile technology. Optimism reflects learners’ perceptions of the advantages of mobile technology (H.-H. Lin et al., 2016). By incorporating all these perceptions and skills, learners are able to actively create and execute their learning plans (B. Lin & Hsieh, 2001).

Previous studies have found e-learning readiness to be associated with engagement, satisfaction, and persistence in online learning (S.-C. Chen et al., 2013; Ji et al., 2022; Prasetya et al., 2021; Zou et al., 2022; Kim et al., 2019; Kumar, 2021). Since mobile learning originates from e-learning and online learning (Ozuorcun & Tabak, 2012), it is very plausible that mobile readiness, like online readiness, would have a similar influence on engagement, satisfaction, and persistence (H.-H. Lin et al., 2016).

### 2.4. Teacher Support

According to Garrison and Cleveland-Innes (2005), interactions between teachers and learners influence learner satisfaction and achievement more strongly than interactions between learners in online learning. Similarly, in the context of mobile-assisted self-directed language learning, teachers are considered to play a critical role (C. Lai, 2015; Y. Lai et al., 2022a). They are expected to provide recommendations and guidance about specific mobile applications, learning materials and learning tips, and encouragement in the learning process to improve students’ learning experience, further leading to effective learning (e.g., García Botero et al., 2019; Hoi & Mu, 2021). Unfortunately, research has found that teachers tend to perceive themselves as having a limited influence on students’ autonomous learning outside the classroom (Chan, 2003; Toffoli & Sockett, 2015). Thus, teachers’ roles should be considered and empirically examined in the discussion of learners’ autonomous learning behaviors outside the classroom (See also C. Lai, 2015).

Previous research has reported the relationship between the teacher’s role and engagement, satisfaction and persistence in various educational contexts (Caskurlu et al., 2020; Hart, 2012; Joo et al., 2013). However, mixed results were found in the relationship between teacher support and engagement in online learning. Jung and Lee (2018) revealed a positive relationship between teaching presence and learning engagement in MOOCs. Yet, Han et al. (2021) found a negative relationship between emotional engagement and teacher support and indicated that Chinese EFL learners who perceived more teacher support were less likely to enjoy online learning. Xu et al. (2020) suggested that teacher facilitation had a positive impact on students’ behavioral and cognitive engagement but no influence on emotional engagement in WeChat-based online semi-synchronous discussions. In addition, C. Lai et al. (2016) revealed various aspects in which students expected their teachers to assist them in improving their knowledge and skills in autonomous language learning with technology outside the classroom. These aspects included metacognitive tips (e.g., how to locate, select and use learning materials) to improve students’ self-directed learning ability, teachers’ in-class technology use to make students familiar with mobile technology and more confident about their own capability to use the technology by themselves, and teachers’ encouragement to enhance students’ perceived advantages from using mobile technology. Based on these results, we could say that teachers’ support could be conducive to students’ self-directed learning, self-efficacy, and optimism, which are the three components of mobile readiness. Hence, we assumed that teacher support is related to mobile readiness as well as satisfaction and persistence.

### 2.5. Language Proficiency

The significance of learners’ language proficiency in online language learning has been underscored by M.-P. Chen et al. (2022) and Chung and Ahn (2022). M.-P. Chen et al. (2022), for example, examined the impact of English proficiency on junior high school students’ English learning attitude, motivation and effectiveness in augmented reality-enhanced contextualized learning. They showed that English proficiency significantly influenced knowledge comprehension. Students with higher English proficiency levels exhibited a more positive attitude towards putting effort into language learning, regulated learning, English, and foreign language learning but less learning anxiety. They also displayed more motivation in terms of self-efficacy, proactive learning and learning value.

### 2.6. This Study

This study aimed to examine how language learners’ persistence and satisfaction were explained by mobile readiness (self-directed learning, optimism, and mobile self-efficacy), teacher support, and engagement in SDLLMT. It also sought to investigate whether differences in SDLLMT existed between students with high and low language proficiencies. The findings may provide implications for enhancing the engagement, satisfaction, and persistence of self-directed learners, thereby facilitating more effective and successful autonomous language learning with technology.

**Figure 1:**
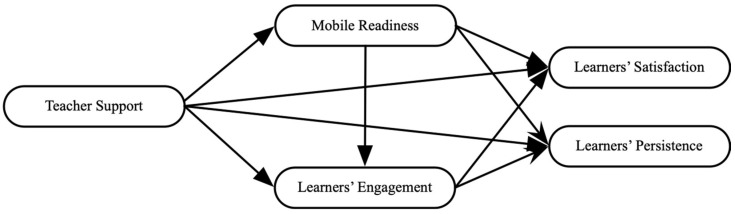
The conceptual model.

Based on the literature review, the following model was proposed as the framework that guided this study (Figure 1).

This study addressed the following research questions:

- Is there any difference in SDLLMT between students with high and low language proficiency?
- How is learners’ satisfaction explained by teacher support, learners’ mobile readiness and engagement in SDLLMT?
- How is learners’ persistence explained by teacher support, learners’ mobile readiness and engagement in SDLLMT?
- How do mobile readiness and engagement mediate the relationship between teacher support and both outcome variables of SDLLMT?

## 3. Methods

### 3.1. Participants and Procedure

In this study, a multidisciplinary sample of volunteers consisted of 446 self-directed English learners from Chinese universities. They were screened based on their response to the first item in the survey (“Do you have any experience learning English by yourself?”). The convenience sampling method was employed to gather data. An anonymous link to the online survey powered by Qualtrics was created to encourage participants to share their preferences. The link was disseminated through social media channels to reach the largest audience possible, targeting students from multiple universities within the primary author’s network. Furthermore, the hyperlink was also shared with university teachers to be sent out to their students. To motivate self-directed learners to complete the survey, we introduced a limited lucky draw as a reward and highlighted the voluntary and enjoyable nature of the survey. We informed participants of the purpose of the survey and how their data is used and asked for their consent at the beginning of the survey. Completing the survey took participants around 5–10 min. Research clearance was obtained from the ICLON Research Ethics Committee.

The data were collected from August to November 2021. A total of 446 respondents visited the questionnaire website, and 352 successfully completed the questionnaire (completion rate was 78.9%). Among the completed questionnaires, there were 28 respondents who stated that they had no experience of self-directed English learning; this left a total of 324 observations making up this study. Demographic information is shown in Table 1.

### 3.2. Instruments

All the instruments used in this study came from existing validated scales. The questionnaire includes the following variables, namely satisfaction and persistence towards SDLLMT adapted from W.-S. Lin and Wang (2012), engagement adapted from Deng et al. (2020), teacher support adapted from Hoi and Mu (2021), mobile readiness (i.e., optimism, self-efficacy, and SDL disposition) adapted from H.-H. Lin et al. (2016), and the demographic characteristics of the students (gender, English exams passed, discipline, educational level). A 5-point Likert scale was used to measure the items, with “1” representing “strongly disagree” and “5” indicating “totally agree”. To ensure responses reflected learner-initiated and learner-controlled contexts, all items were preceded by the following: “When self-studying English outside of class…”. The phrase “self-studying” signaled voluntary initiation, while “outside of class” emphasized learner control over time, place, and content. This framing oriented participants toward self-directed learning’s core features, strengthening construct validity. Since the instruments are in English, all the items were translated into Chinese by the first author and then translated back by other bilingual teachers to ensure semantic accuracy and equivalence. To ensure content validity, the questionnaire was sent to two academic professors for internal review and ten university students for a pilot test. Based on their feedback and suggestions, we modified the wordings to prevent semantic bias.

### 3.3. Data Analysis

Four stages of analyses were performed. Firstly, an independent sample *t*-test was used to examine whether students with high and low language proficiency differed in their mobile readiness, engagement, satisfaction, persistence, and the teacher support they received in SDLLMT. The results showed that the two groups showed no statistical difference in terms of learners’ mobile readiness (t = −0.531, *p* > 0.05), engagement (t = −1.273, *p* > 0.05), satisfaction (t = −0.857, *p* > 0.05), persistence (t = −1.224, *p* > 0.05), and teacher support they received (t = −1.131, *p* > 0.05). Therefore, language proficiency was not included in further analyses.

Secondly, based on the proposed model, we adopted structural equation modeling to test these relationships. The measurement model was estimated with confirmatory factor analysis to evaluate the extent to which the observed items gauged the latent constructs. Composite reliability (CR), Cronbach’s alpha, and average variance extracted (AVE) were used to test the model’s reliability and convergent validity. To determine discriminant validity, the method using the heterotrait–monotrait (HTMT) ratio of correlations, a new criterion, was employed. Specifically, the HTMT is calculated from the average of the heterotrait–hetero method correlations relative to the average of the monotrait–hetero method correlations (Tang et al., 2021). The elements of the HTMT are given by the equation below.

$$HTMT_{ij}=\frac{\frac{1}{K_{i}K_{j}}\sum_{g=1}^{K_{i}}\;\sum_{h=1}^{K_{j}}\;r_{ig,jh}}{{\left(\frac{2}{K_{i}\left(K_{i}-1\right)}\cdot \sum_{g=1}^{K_{i}-1}\;\sum_{h=g+1}^{K_{i}}\;r_{ig,ih^{*}}\frac{2}{K_{j}\left(K_{j}-1\right)}\cdot \sum_{g=1}^{K_{j}-1}\;\sum_{h=g+1}^{K_{j}}\;r_{jg,jh}\right)}^{\frac{1}{2}}}$$

$K_{i}$ and $K_{j}$ denote the number of items of constructs i and j, respectively.

Thirdly, the structural model was used to estimate the relationships among latent constructs. The chi-square to degrees of freedom (<3), Tucker–Lewis index (>0.9), root mean square error of approximation (<0.8), comparative fit index (>0.9) and standardized root mean square residual (<0.8) were used to determine model fitness. Fourthly, we performed a mediation analysis using a bias-corrected bootstrapping of 5000 samples (Preacher & Hayes, 2008).

## 4. Results

### 4.1. Measurement Validation

Table 2 summarizes information on reliability and convergent validity. All the items ranged from 0.643 to 0.857, which are above the recommended threshold value of 0.5 (Fornell & Larcker, 1981). Internal consistency reliability was estimated by CR and Cronbach’s alpha. The recommended threshold of these two indicators is 0.7 (Hair et al., 2006). Based on Table 1, all the constructs exceeded 0.7, suggesting good internal consistency. The AVE values of all constructs were greater than 0.5 (from 0.50 to 0.69), indicating the satisfactory convergent validity of the constructs (Fornell & Larcker, 1981). Concerning discriminant validity, the HTMT value between two factors should ideally be below 0.85 (Kline, 2015), but it can go up to 0.90 if the constructs are conceptually similar (Yusoff et al., 2020). The HTMT values of the three subconstructs of engagement did not exactly meet the suggested criteria. In order to address the problem of low discriminant validity among the three subconstructs, we combined these subconstructs into one overall measure, and only three items were left (two from cognitive engagement and one from emotional engagement). Hence, all the HTMT values were below 0.85, which met the suggested criteria of discriminant validity, as illustrated in Table 3.

To avoid multicollinearity, common method bias was assessed using Harman single-factor analysis, because inflating correlations between variables is a potential threat (MacKenzie & Podsakoff, 2012). Podsakoff et al. (2003) suggested that if the proportion of a single factor explaining the total variance is below 50%, common method bias does not exist. Our results show that the highest variance for a single factor was 38.3%, which indicates that common method bias was not a concern in the current study.

### 4.2. Test of Structural Model

**Figure 2:**
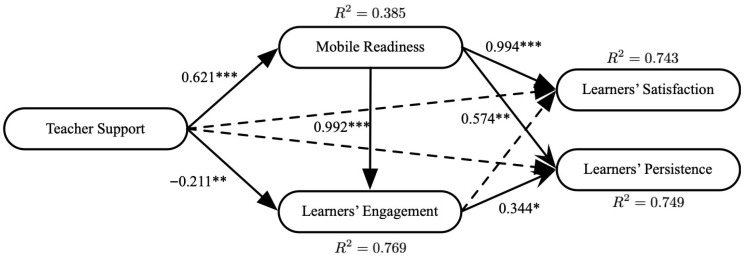
Results. * *p* < 0.05, ** *p* < 0.01, *** *p* < 0.001.

We analyzed the structural model fit using maximum likelihood estimation. As illustrated in Table 4, the structural model indicated a good fit with the collected data, meeting the criteria suggested by Kline (2015). The proportions of explained variance were 38.5% for mobile readiness, 76.9% for engagement, 74.3% for satisfaction, and 74.9% for persistence. Figure 2 shows the relationships between variables.

Teacher support (β = 0.621, *p* < 0.001) had a significant effect on mobile readiness. Mobile readiness (β = 0.992, *p* < 0.001) and teacher support (β = −0.211, *p* < 0.01) were significantly associated with engagement in SDLLMT. Mobile readiness (β = 0.994, *p* < 0.001) was directly related to satisfaction. Mobile readiness (β = 0.574, *p* < 0.01) and engagement (β = 0.344, *p* < 0.05) had a direct relationship with persistence in SDLLMT.

### 4.3. Mediation Analysis

To examine the mediating effect, we verified the statistical significance of the indirect effect by conducting a bias-corrected bootstrapping of 5000 samples. Bootstrapping has been widely used to test whether a mediating variable carries the significant influence of an independent variable onto a dependent variable (Preacher & Hayes, 2008). A mediation relationship was found in this study. The effect of teacher support on engagement was significantly mediated by mobile readiness (β = 0.616, *p* < 0.001, 95%CI [0.407; 0.896]). The other mediation effects were not significant.

## 5. Discussion

This study examined the influence of mobile readiness and teacher support on learners’ persistence, satisfaction, and engagement in SDLLMT. Teacher support was significantly and positively associated with learners’ mobile readiness but negatively with learners’ engagement. Mobile readiness made a significant and positive contribution to learners’ engagement, satisfaction, and persistence. Learners’ engagement was significantly and positively related to persistence. Additionally, mobile readiness mediated the link between teacher support and engagement. These main findings are discussed below.

### 5.1. Relationship Between Teacher Support, Learners’ Mobile Readiness, and Engagement

Teacher support was significantly and positively linked to learners’ mobile readiness. The significant and positive relationship between the two variables provides empirical evidence that teachers can assist students in improving their self-directed learning skills and enhancing their perceived capability of using mobile technology and their perception of the advantages of mobile technology. Several studies (e.g., Hoi & Mu, 2021; C. Lai, 2015) support the idea that affective support from teachers can influence learners’ perception of the advantages of technology in language learning. Yet, little empirical research has been done on teacher influence on self-directed learning skills and the perceived capability of using mobile technology.

Learners’ mobile readiness was found to make a strong contribution to engagement in SDLLMT, backing up the finding of Kim et al. (2019), indicating a positive and significant relationship between digital readiness and engagement in an e-learning environment. Since self-directed learning using mobile technology outside class is completely up to learners (Y. Lai et al., 2022a), they have absolute learning autonomy. This means that learners’ characteristics will exert a great influence on their engagement (Kuo et al., 2021). It is a reasonable assumption that learners who are well-prepared for this mobile learning are more likely to be engaged in the learning process. More specifically, learners with higher mobile readiness, including stronger self-directed learning skills, positive perceptions about the affordances of mobile technology in self-directed language learning, and greater confidence in using it, tend to remain engaged throughout the learning process.

Teacher support was significantly yet negatively related to learners’ engagement, but it showed an indirect and positive impact on learners’ engagement with a mediating role in mobile readiness. Despite the mixed findings of the direct relationship between teacher support and learners’ engagement reported in the literature review, a possible explanation for the negative relationship between teacher support and engagement is that students in student-initiated and -controlled learning environments might be more likely to enjoy the feeling of fully controlling their own learning process and might be unhappy with a lot of teacher involvement. From the perspective of Self-Determination Theory (SDT; Ryan & Deci, 2000), the quality of teacher support matters critically. SDT distinguishes between autonomy-supportive teaching (which facilitates engagement) and controlling teaching (which undermines it). If students perceive teacher support as intrusive monitoring or pressure to conform, rather than as encouragement for self-directed exploration, it may frustrate their basic need for autonomy, thereby reducing intrinsic motivation and engagement (Reeve, 2009). In addition, from a cultural perspective, in China, teachers are still quite important for learners, even in this self-directed and out-of-class context. Direct teacher involvement might put mental or emotional pressure on self-directed learners, further decreasing their engagement in the learning process. The mediating role of mobile readiness might be understood considering that learners receiving more teacher support had higher mobile readiness, which in turn led them to be more engaged in SDLLMT. Although many self-directed learners might prefer not to have teacher support in SDLLMT, quite a few of them might not be well enough prepared to proceed successfully on their own. They still need teachers to lead them in the self-directed learning journey. For example, teachers could inform them about the affordances and possibilities of mobile technology in language learning (optimism towards mobile technology) and give them metacognitive tips on how to learn by themselves (self-directed learning skills) and how to effectively utilize technology (self-efficacy of using mobile technology) (Carson & Mynard, 2012; K. Gray et al., 2010), eventually enhancing their mobile readiness. Equipped with increased mobile readiness, students could possibly engage in this learning process more because they would feel a sense of achievement (Tsay & Brady, 2010). Rather than viewing support and autonomy as opposing forces, we propose teacher support as autonomy-enabling. Through enhancing students’ mobile readiness, teachers indirectly foster engagement and outcomes. This extends theory by revealing how external support equips learners to exercise autonomy more effectively, demonstrating that teachers remain consequential even in self-directed contexts.

### 5.2. Factors Related to Learners’ Satisfaction and Persistence

Mobile readiness significantly and positively predicted learners’ satisfaction and persistence, echoing the claims of Ji et al. (2022), Kumar (2021), Wei and Chou (2020), and S.-C. Chen et al. (2013), demonstrating a positive relationship between online readiness/technology readiness and students’ satisfaction and continuance intention in online learning. Regarding satisfaction, it can be inferred from our findings that learners are likely to feel more satisfied if they are better prepared when they start SDLLMT (i.e., greater self-directed learning skills, more optimistic perception of the advantages of and their ability to use mobile technology). Other researchers have shown partially significant relationships between mobile readiness and persistence. S.-C. Chen et al. (2013) found that technology readiness exerted a positive influence on users’ persistence with mobile services. Yet, Leung and Chen (2019) reported that innovation, one of the drivers of technology readiness, was a significant predictor of continuance intention, whereas optimism, another driver, was not. Given the unclear relationship between mobile readiness and persistence and lack of research into mobile readiness, further research should pay attention to this aspect.

Teacher support was not found to significantly influence either learners’ satisfaction or persistence in SDLLMT, which contradicts the findings of previous studies (e.g., Caskurlu et al., 2020; J. C. Yang et al., 2016), which revealed significant and positive relationships between teaching presence and students’ satisfaction and persistence in online courses. In learner-initiated and -directed learning environments, as claimed above, learners might enjoy the feeling of fully controlling their own learning process and feel unease and unhappiness with a great deal of teacher involvement. Meanwhile, because of the emotional pressure from Chinese teachers with high authority (Guo & Xu, 2021), learners may not be willing to directly ask for teachers’ help and guidance. Hence, future studies need to figure out the in-depth relationships among these variables in the context of self-directed learning.

Learners’ engagement was not related to satisfaction but had a direct effect on persistence. The non-significant relationship between engagement and satisfaction is surprising since other studies have found that learners’ engagement could predict satisfaction (Fisher et al., 2021; Rajabalee & Santally, 2020). As mentioned earlier in Section 4.1, we initially used three dimensions of learners’ engagement but eventually combined these subconstructs into one overall measure. More importantly, Lane et al. (2021) and B. W. Gao et al. (2020) both proved that cognitive engagement failed to explain learners’ satisfaction in blended learning. Specifically, B. W. Gao et al. (2020) indicated that students were satisfied only when they were emotionally and fully engaged as emotional engagement had a positive impact on satisfaction, but cognitive engagement did not. Lane et al. (2021) also showed that emotional engagement was the most frequent predictor of student satisfaction in all four courses they studied, namely Human Geography, Math, Chemistry, and computing science, and cognitive engagement only explained satisfaction in the computing science course. We therefore assume that the non-significant relationship between engagement and satisfaction that we found could be caused by two items of cognitive engagement. More research is needed to examine the relationships between the subdimensions of engagement and satisfaction and to further uncover explanations for these relationships through interview analysis. In addition, the significant relationship between engagement and persistence found in this study coincides with the findings of Jung and Lee (2018), which indicated the direct and positive effect of learning engagement on persistence in MOOCs.

## 6. Limitation and Future Research

Some shortcomings of the current study should be mentioned here. Firstly, this study was based on learners’ self-reported scales of engagement, satisfaction, and persistence. Future research could use recorded data to track learners’ engagement and persistence and recorded comments and reviews to extract learners’ emotional perception through sentiment analysis techniques as indicators of satisfaction. Learning analytics could be used to explain learners’ behavior based on large amounts of learning data. Additionally, qualitative methods could also be employed to examine students’ learning process and performance in further research. In this way, additional information could be collected about the factors that are related to the efforts students put in language learning with mobile technology. This additional information might provide more specific implications regarding how to improve students’ learning. Secondly, along with learners’ ever-changing mobile readiness and persistence usage, the cross-sectional nature of this study did not allow for conclusions about students’ development in persistence and satisfaction over time. A longitudinal design could be encouraged in subsequent studies. Thirdly, the language proficiency level was based on English examinations that Chinese students have passed. However, these examinations may not accurately classify students with low-level or high-level language proficiency, because only language-major students are required to take Tests for English Major 4 and 8. More rigorous measures of language proficiency should be encouraged to investigate whether language proficiency levels indirectly or interactively affect students’ SDLLMT. Fourthly, due to little empirical research on teacher influence on self-directed learning skills and the perceived capability of using mobile technology, we call for more empirical studies to further strengthen the findings of our study. Fifthly, this study measured engagement as a single-dimension construct using three items to ensure discriminant validity. While statistically convenient, this approach limited theoretical depth. Future research should adopt multidimensional scales to explore the distinct roles of behavioral, emotional, and cognitive engagement. Longitudinal designs and qualitative methods like learner diaries are recommended to capture engagement’s evolving nature and mitigate common method bias. And this paper includes teachers’ and learners’ perspectives in the process but not the technology perspective. Technological features such as system quality, information quality, and service quality have been found to have significant influences on the learning process and success (Al-Fraihat et al., 2020). It would be worthwhile to exploit this gap by integrating these variables into the model to yield a better understanding of SDLLMT. Finally, the theoretical mechanisms underlying our model warrant further investigation. Future research could draw on established psychological or educational theories to examine the finer-grained processes through which teacher support influences mobile readiness, engagement, and learning outcomes.

## 7. Conclusions

This study identified the impact of learners’ mobile readiness, teacher support, and engagement on learners’ satisfaction and persistence. Teacher support was found to be significantly and positively related to learners’ mobile readiness but negatively to learners’ engagement. Mobile readiness made a significant and positive contribution to learners’ engagement, satisfaction, and persistence. Learners’ engagement was also significantly and positively related to persistence. Furthermore, mobile readiness mediated the link between teacher support and engagement.

A unique contribution of the present research is that it was one of the first attempts to identify the determinants of persistence and satisfaction from the perspectives of language learners in SDLLMT. As self-directed, informal language learning remains a relatively under-researched area in the field of MALL (Kukulska-Hulme, 2016, p. 138), this study expanded the existing body of literature by putting forth and verifying a conceptual model of learners’ persistence and satisfaction building upon antecedent studies. The comprehensive model offers a better understanding of the mechanism of how learners’ mobile readiness and teacher support relate to learners’ satisfaction and persistence outside the classroom. Secondly, although readiness has been extensively investigated over the years in the field of online learning and e-learning, empirical studies have rarely introduced it as a construct in mobile learning and the post-acceptance phase (Leung & Chen, 2019). Due to the distinctions between mobile readiness and online learning/e-learning readiness, as explained in Section 2, this empirical study firstly encompassed and confirmed mobile readiness as a construct in the context of mobile learning and self-directed learning to open a discussion about future learning initiatives.

In practical terms, considering the importance of learners’ mobile readiness and the critical impact of teacher support on it in informal learning, teachers may influence learners’ self-directed learning skills and their perception of the advantages and their own capability of using mobile technology in self-directed language learning. More precisely, teachers could foster learners’ self-directed learning skills by offering them opportunities to gradually direct their own learning processes (Francom, 2010). Teachers might also help learners understand the advantages of mobile technology by designing mobile-based activities to emphasize the positive functions of mobile technology in the foreign language class and then encouraging them to extend this to out-of-class and self-directed learning. Furthermore, teachers can enhance learners’ capability in using mobile technology by offering students technical guidance, recommendations on useful online language resources and explicit demonstrations of how to use these resources effectively (Hoi & Mu, 2021; Morris & Rohs, 2021). The direct and indirect effects of mobile readiness highlight its crucial role in mobile learning. Self-directed learners should attach great significance to their own mobile readiness. Based on our results, learners with higher self-directed learning skills and those who perceive the advantages of mobile technology and feel confident in using it tend to feel satisfied and persist in the process of SDLLMT, resulting in effective and successful learning.

## Figures and Tables

**Table 1:** Frequency and percentage of participants by gender, education, and major.

Demographic Factors	*N*	%
Gender		
Male	156	48.15
Female	168	51.85
Education		
Undergraduate	248	76.54
Postgraduate	76	23.46
Major		
Language	108	33.33
Non-language	216	66.67
English Language proficiency ^1^		
Low level	121	37.35
High level	203	62.65

^1^ Regarding language proficiency, students who had only passed College English Test 4 were coded as low-level, while those who had passed College English Test 6, Test for English Major 4, or Test for English Major 8 were coded as high-level.

**Table 2:** Instrument validity and reliability.

Construct	Sub Construct	Number of Items	Factor Loading	CR	AVE	Cronbach’s α
Teacher support (TS)		6	0.66–0.84 ***	0.89	0.58	0.89
Persistence (LP)		3	0.81–0.85 ***	0.87	0.69	0.87
Satisfaction (LS)		5	0.68–0.78 ***	0.86	0.55	0.86
Engagement (LE)		3	0.71–0.78 ***	0.78	0.55	0.78
Mobile readiness (MR)	SDL disposition	4	0.68–0.83 ***	0.83	0.55	0.83
	Self-efficacy (SE)	7	0.71–0.86 ***	0.93	0.66	0.93
	Optimism (OP)	3	0.64–0.77 ***	0.75	0.50	0.74

Note. *** *p* < 0.001.

**Table 3:** Heterotrait–monotrait (HTMT) between study constructs.

Constructs	TS	LP	LS	LE	SDL	SE	OP
TS							
LP	0.476						
LS	0.556	0.725					
LE	0.427	0.822	0.727				
SDL	0.626	0.529	0.721	0.612			
SE	0.429	0.724	0.573	0.696	0.476		
OP	0.482	0.686	0.765	0.704	0.704	0.672	

**Table 4:** Model fit statistics.

	χ^2^	df	χ^2^/df	CFI	TLI	RMSEA	SRMR
Structural model	879.221	422	2.08	0.926	0.918	0.058	0.062
Criteria (Browne et al., 1993)			<3	>0.9	>0.9	<0.08	<0.08

## Data Availability

Data will be made available on request.

## Abbreviations

The following abbreviations are used in this manuscript:

SDLLMT	Self-directed language learning using mobile technology
